# One-Step Synthesis of Novel Renewable Vegetable Oil-Based Acrylate Prepolymers and Their Application in UV-Curable Coatings

**DOI:** 10.3390/polym12051165

**Published:** 2020-05-19

**Authors:** Yupei Su, Hai Lin, Shuting Zhang, Zhuohong Yang, Teng Yuan

**Affiliations:** 1 Guangdong Laboratory of Lingnan Modern Agricultural Science and Technology, Key Laboratory for Biobased Materials and Energy of Ministry of Education, College of Materials and Energy, South China Agricultural University, Guangzhou 510642, China; suyupei@stu.scau.edu.cn (Y.S.); linhai@stu.scau.edu.cn (H.L.); stzhang@scau.edu.cn (S.Z.); 2 Department of Applied Physics, The Hong Kong Polytechnic University, Hung Hom, Kowloon 999077, Hong Kong, China

**Keywords:** vegetable oil, one-step synthesis, acrylate prepolymers, bio-based materials, eco-friendly, UV-curable coatings

## Abstract

With the rapid development of social economy, problems such as volatile organic compound (VOC) pollution and the excessive consumption of global petroleum resources have become increasingly prominent. People are beginning to realize that these problems not only affect the ecological environment, but also hinder the development of the organic polymer material industry based on raw fossil materials. Therefore, the modification and application of bio-based materials are of theoretical and practical significance. In this study, a series of vegetable oil-based acrylate prepolymers were synthesized by one-step acrylation using palm oil, olive oil, peanut oil, rapeseed oil, corn oil, canola oil, and grapeseed oil as raw materials, and the effect of different double bond contents on the product structure and grafting rate was investigated. Furthermore, the as-prepared vegetable oil-based acrylate prepolymers, polyurethane acrylate (PUA-2665), trimethylolpropane triacrylate (TMPTA), and photoinitiator (PI-1173) were mixed thoroughly to prepare ultraviolet (UV)-curable films. The effect of different grafting numbers on the properties of these films was investigated. The results showed that as the degree of unsaturation increased, the acrylate grafting number and the cross-linking density increased, although the acrylation (grafting reaction) rate decreased. The reason was mainly because increasing the double bond content could accelerate the reaction rate, while the grafted acrylic groups had a steric hindrance effect to prevent the adjacent double bonds from participating in the reaction. Furthermore, the increase in grafting number brought about the increase in the structural functionality of prepolymers and the cross-linking density of cured films, which led to the enhancement in the thermal (glass transition temperature) and mechanical (tensile strength, Young’s modulus) properties of the cured films.

## 1. Introduction

According to different curing temperatures, traditional curing methods can be divided into room temperature curing and heat curing. Curing coatings by heating at high temperature is often time-consuming, energy-intensive, and not suitable for heat-sensitive substrates [1]. Compared with traditional thermosetting coatings, ultraviolet (UV)-curable coatings have developed rapidly due to their advantages of the “5Es” (Efficient, Environmentally friendly, Energy saving, Enabling, and Economical) [2,3]. The above advantages of UV-curable coatings can be specifically explained as follows. First, the UV-curing process can be completed in a few seconds, thereby greatly improving the curing efficiency. Second, reactive diluents that can polymerize with prepolymers to form cured films are usually used to replace common volatile solvents, which reduces the use of organic solvents and exhibits environmentally-friendly properties. Third, UV-curable coatings can be cured at room temperature, avoiding the curing process at high temperatures, thereby saving energy. In addition, UV-curable coatings can be applied to various substrates including heat-sensitive substrates such as plastics and papers. Finally, compared with thermo-curing, the equipment used for UV-curing has a more compact volume, and the curing process exhibits higher efficiency and consumes less energy, which makes the UV-curing process more economical [4,5,6]. Nowadays, UV-curable coatings have been widely used in printing [7,8], packaging [9], electronic products [10,11], communications [12], automotive [13], aerospace [14], and so on, and they can replace traditional thermosetting coatings in certain applications [15]. UV-curable coatings usually consist of different components including photoinitiators, prepolymers, and reactive diluents. The main reaction in UV-curing is that UV irradiation causes the photoinitiators to decompose to form active free radicals, which further initiates the cross-linking polymerization of the prepolymers and the reactive diluents. Although UV-curable coatings have penetrated into every aspect of people’s lives, there are still a series of problems that need to be addressed. For example, the raw materials for UV-curable coatings are all extracted from petrochemical products, whereas the supply of petroleum resources is not stable all over the world. The fact that the raw materials from petrochemical products rely too much on the upstream supply chain, resulting in the unstable supply of raw material and high prices. In addition, petrochemical products are non-renewable, which puts a lot of pressure on environmental regulation. More importantly, large amounts of volatile organic compounds (VOCs) are emitted during the preparation of petrochemical products, which seriously damages people’s health [16,17].

Therefore, in order to overcome the drawbacks of petroleum-based coatings, renewable resources such as vegetable oils [18], cellulose [19], and lignin [16] have begun to attract much attention. Among them, vegetable oils are widely used because of their low price, renewability, and abundance. However, given that the reactivity of various groups in the long chains of most vegetable oils is not high, it is necessary to chemically modify these vegetable oils to introduce more active groups. Lee et al. used the in situ epoxy method to epoxidize soybean oil, and then the ring-opening reaction of epoxy groups was implemented with acrylic acid. The remaining epoxy groups were reacted with triethylenetetramine to prepare a UV-curable soybean oil-based pressure-sensitive adhesive, and the resultant rheology, peeling strength, bond strength, and tensile strength were tested. The pressure-sensitive adhesive could be used to repair the damaged surfaces of chemical containers [20]. Liu et al. carried out a ring-opening reaction of cardanolglycidyl ether with a polyacid, and then epoxidized and acrylated the unsaturated side chain of the cardanol segment to prepare a multi-arm cardanol-based acrylate prepolymer. Compared with acrylated epoxidized soybean oil, the results showed that the cardanol-based coatings exhibited higher hardness (3 H), better adhesion (100%), and higher glass transition temperature (39 °C), and they also have a high bio-based content (59.6%). Due to the unique structure, the prepolymer contained the structural characteristics of a rigid benzene ring, flexible alkyl long chain, and polar hydroxyl group, which enhanced the performance of the films [21]. Liang et al. used castor oil and mercaptoacetic acid as raw materials to prepare mercapto castor oil by the thiol-ene reaction. Afterward, glycidyl methacrylate was used to prepare castor oil-based prepolymers via esterification. The as-prepared UV-cured films showed high pencil hardness (5–6 H) and good acid (alkali) resistance [22]. Liang et al. performed the Diels–Alder reaction between tung oil and maleic anhydride to obtain tung-maleic anhydride, then reacted it with epichlorohydrin to introduce epoxy groups, and finally pentaerythritol triacrylate was used to react with the epoxy groups to obtain multifunctional tung oil-based prepolymers. The prepared UV-cured films had excellent hardness (4–6 H), high glossiness (127.6–173.2), and excellent water resistance [18]. Wang et al. prepared castor oil-based mercaptan by the esterification of castor oil and mercaptoacetic acid, which was then mixed with phosphorus and silicon-containing reactive diluents to prepare UV-curable flame retardant coatings. It was found that the flame retardant treated wood had a limited oxygen index (LOI) of 27.52%, which effectively reduced the peak heat release rate and smoke production rate, and the char yield could reach 14.41% [23]. Hu et al. used acryloyl chloride and cardanol hydroxyl to prepare monofunctional UV-curable reactive diluents, which were further mixed with a castor oil-based prepolymer to prepare UV-curable films. In comparison to petroleum-based reactive diluent 2-hydroxyethyl acrylate, the use of a cardanol-based reactive diluent facilitated the increase in thebio-based content of the films, reducing the viscosity and volume shrinkage of the systems [24]. Chen et al. synthesized a UV-curable castor oil-based polyurethane acrylate (COME-PUA) through photo-click chemistry and the isocyanate polyurethane reaction. First, β-mercaptoethanol (ME) was chemically grafted to castor oil (CO) by the thiol-ene photo-click reaction. The double bonds in CO were almost completely converted within 10 min, and the average hydroxyl functionality of the resulting polyol COME was about 6. Afterward, COME and CO were modified by the isocyanate polyurethane reaction with hydroxypropyl acrylate (HPA) to prepare COME-PUA and CO-PUA. The results showed that COME-PUA with higher functionality had a higher reaction rate and pencil hardness than CO-PUA [25]. Different bio-based materials could be obtained by modifying vegetable oils. By continuously expanding their application fields, it is possible for vegetable oils to replace some conventional energy sources. Existing modification methods often require two or more steps of reaction under harsh and complicated reaction conditions; however, a low product yield was achieved. This challenges researchers to develop new and efficient modification technologies to tap the potential value of vegetable oils. The one-step acrylation reaction has the advantages of simple operation, mild conditions, and atom economy, and it is an ideal method for modifying vegetable oils. Progress has been made in adding acrylic acid groups directly to the carbon–carbon double bonds of vegetable oils. Eren et al. have conducted a lot of research on this type of reaction and used castor oil, N-bromosuccinimide, and acrylic acid as raw materials to prepare brominated acrylate castor oil in one step, then reacted it with toluene diisocyanate to prepare a castor oil-based polyurethane [26]. They also prepared bromoacrylated methyl oleate using this method [27]. In addition, in order to obtain acrylamide derivatives of sunflower oil, they also used the Ritter reaction, but the reaction was carried out at −20 °C using concentrated sulfuric acid as thecatalyst [28]. Walther et al. mixed alkyd resin with acrylic acid and N-bromosuccinimide, and the mixture was reacted at room temperature for seven days to obtain a bromoacrylated alkyd resin [29]. Zhang et al. carried out a one-step acylation of acrylic acid and soybean oil under different catalysts to explore the effect of different catalysts on the reaction process. The products were thermocured with styrene to obtain thermosetting films. The results showed that as the acrylation degree increased, the thermosetting films became harder [30,31]. The above studies indicated that the one-step reaction was restricted by the reactants and the catalysts. Thus, highly active reactants and highly efficient catalysts are the key to improving the application value of the one-step reaction.

In this paper, different vegetable oil-based acrylate prepolymers were prepared through one-step acrylation. The chemical structures of the products were determined by ^1^H NMR and FT-IR. Different vegetable oil-based acrylate prepolymers were mixed with polyurethane acrylate (PUA-2665), trimethylolpropane triacrylate (TMPTA), and photoinitiator PI-1173 to prepare UV-curable films. Dynamic thermo-mechanical analysis (DMA), thermo-gravimetric analysis (TGA), and tensile tests were used to characterize their properties, which was also used to analyze the influence of the grafting number and chemical structure on the properties of the UV-curable films.

## 2. Materials and Methods

### 2.1. Materials

**Scheme 1:**
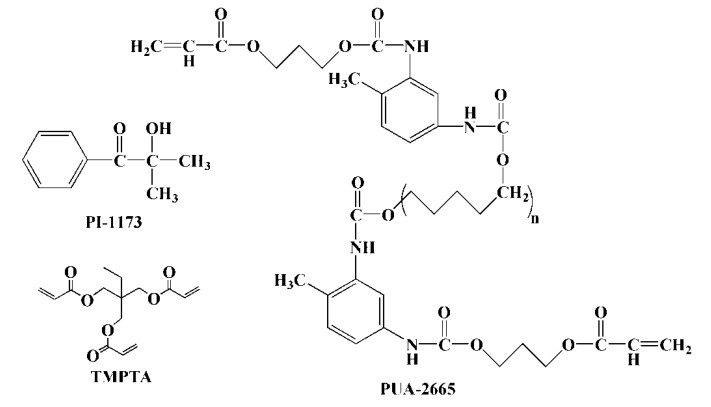
Chemical structures of PI-1173 (2-hydroxy-2-methyl propiophenone), TMPTA (trimethylolpropane triacrylate), and polyurethane acrylate (PUA-2665).

Canola oil (CaO, chemical pure) was purchased from Ji’an Zhongxiang Natural Plant Co. Ltd. (Ji’an, China). Rapeseed oil (RSO, chemical pure) was purchased from Guangzhou Runquan Chemical Co. Ltd. (Guangzhou, China). Grapeseed oil (GSO, chemical pure) was purchased from Guangzhou Yazhi Cosmetics Co. Ltd. (Guangzhou, China). Peanut oil (PeO, chemical pure) was purchased from Shandong Luhua Marketing Co. Ltd. (Yantai, China). Palm oil (PaO, chemical pure) was purchased from Guangzhou Duode Chemical Co. Ltd. (Guangzhou, China). Olive oil (OO, analytical pure) and corn oil (CoO, analytical pure) was purchased from Shanghai Maclean Biochemical Technology Co. Ltd. (Shanghai, China). Acrylic acid (AA, analytical pure), *n*-hexane (analytical pure), sodium bicarbonate (analytical pure), and anhydrous magnesium sulfate (analytical pure) were obtained from Tianjin Fuchen Chemical Reagent Co. Ltd. (Tianjin, China). Boron trifluoride diethyl ether solution (BF_3_, 46.5%) was bought from Shanghai Lingfeng Chemical Reagent Co., Ltd. (Shanghai, China). Polyurethane acrylate (PUA-2665, chemical pure, with a molecular weight of 3000 g·mol^−1^ and a viscosity of 1000 mPa·s) was supplied by Zhaoqing Power Dream Chemical Co. Ltd. (Zhaoqing, China) [32]. Trimethylolpropane triacrylate (TMPTA, chemical pure) and 2-hydroxy-2-methylpropiophenone (photoinitiator, denoted as PI-1173, chemical pure) were received from BASF (China) Co. Ltd. (Shanghai, China). The chemical structures of PI-1173, TMPTA, and PUA-2665 employed in this work are shown in Scheme 1. All of the above chemical reagents were used without further treatment.

### 2.2. Synthesis of Vegetable Oil-Based Acrylate Prepolymers

**Scheme 2:**
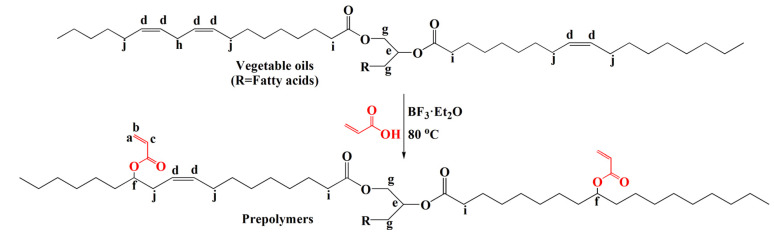
Synthetic route to vegetable oil-based acrylate prepolymers.

As shown in Scheme 2, certain amounts of vegetable oils, acrylic acid, and boron trifluoride ether solution were added in a 500 mL single-necked flask. The molar ratio of the carbon–carbon double bond to acrylic acid was1:4, and the molar ratio of the carbon–carbon double bond to boron trifluoride ether was 1:0.6. The mixture was reacted at 80 °C under stirring for 2 h. Afterward, the mixture was poured into a separating funnel and extracted with *n*-hexane. A saturated aqueous solution of sodium bicarbonate was added to remove the unreacted acrylic acid and boron trifluoride ether solution. The cessation of bubbles indicated that the excess reactants had been washed away. The upper orange-yellow organic phase was transferred to a beaker, and an appropriate amount of anhydrous magnesium sulfate was added to dry overnight and then filtered. Finally, the solvent was removed by vacuum distillation and dried to obtain a yellow liquid vegetable oil-based acrylate prepolymer. Vegetable oil-based acrylate prepolymers prepared from palm oil (PaO), olive oil (OO), peanut oil (PeO), rapeseed oil (RSO), corn oil (CoO), canola oil (CaO), and grapeseed oil (GSO) were denoted as APaO, AOO, APeO, ARSO, ACoO, ACaO, and AGSO, respectively.

### 2.3. Preparation of UV-Curable Films

Different vegetable oil-based acrylate prepolymers obtained above were mixed with polyurethane acrylate (PUA-2665), trimethylol propanetriacrylate (TMPTA), and photoinitiator (PI-1173). The mass fraction of vegetable oil-based acrylate prepolymers was 50%, the mass fraction of TMPTA was 20%, the mass fraction of PUA-2665 was 27%, and the mass fraction of PI-1173 was 3%. The mixtures were poured on the surface of tinplates, and the wet film samples were prepared by using a 500 μm wet film coater. Thereafter, the wet films were placed under a 365 nm UV-LED light (the radiation intensity was about 378.3 mW·cm^−2^) and irradiated for 10 s to obtain UV-cured films.

### 2.4. Characterization

The functional groups of the samples were analyzed using Thermo-Nicolet iS10 spectrometer (Thermo Fisher Scientific Inc., Waltham, MA, USA). The scanning measurement range of Fourier transform infrared (FT-IR) spectra was 4000 to 400 cm^−1^.

The proton nuclear magnetic resonance (^1^H-NMR) characterization of the samples was performed using a Bruker AV600spectrometer (Bruker Corporation, Billerica, MA, USA). Tetramethylsilane (TMS) was used as the internal standard, and deuterated chloroform (CDCl_3_) was used as the solvent to analyze the molecular structure of the samples.

A DMA 242E dynamic thermo-mechanical analyzer (Erich NETZSCH GmbH & Co. Holding KG, Selb, Bavaria, Germany) was used to analyze the dynamic thermo-mechanical properties of the samples. The tensile bracket was selected, the sample size was 20.0 mm (length) × 6.0 mm (width) × 0.5 mm (thickness), and the oscillation frequency was 1 Hz. Samples were first cooled to −80 °C with liquid nitrogen for 3 min, then the temperature was increased to 180 °C at a rate of 5 °C·min^−1^. Glass transition temperature (*T*_g_) of the cured films was obtained from the peak of the tanδ curves.

Mechanical properties of the samples were studied employing a Shimadzu AGS-X 1 kN universal testing machine (Shimadzu Corporation, Kyoto Prefecture, Kyoto, Japan). Tensile bracket was selected, the size of the samples was 40.0 mm (length) × 10.0 mm (width) × 0.5 mm (thickness), and the crosshead speed was 10 mm·min^−1^. For accuracy, each sample was measured three times.

The Netzsch STA 449C thermo-gravimetric analyzer (Erich NETZSCH GmbH & Co. Holding KG, Selb, Bavaria, Germany) was used to investigate the thermal stability of the samples. Under the protection of nitrogen at a flow rate of 60 mL·min^−1^, the temperature of the samples increased from 35 to 650 °C at a rate of 10 °C·min^−1^.

## 3. Results and Discussion

### 3.1. Structure Analysis of Vegetable Oil-Based Acrylate Prepolymers

As the FT-IRand ^1^H NMR spectra of all vegetable oil-based acrylate prepolymers were similar, corn oil (CoO) and corn oil-based acrylate prepolymer (ACoO) were selected as representative examples.

**Figure 1:**
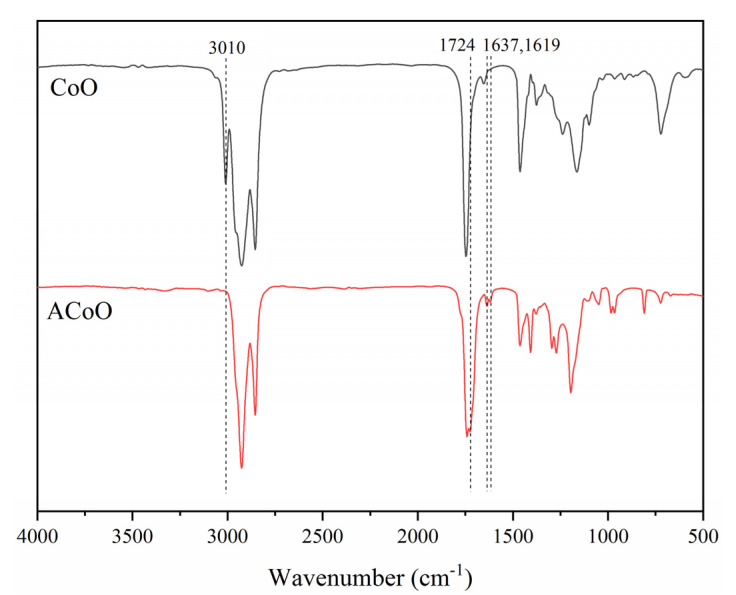
Fourier transform infrared (FT-IR) spectra of corn oil (CoO) and corn oil-based acrylate prepolymer (ACoO).

The FT-IR spectra of CoO and ACoO are shown in Figure 1. In the spectrum of CoO, the peak at 3010 cm^−1^ corresponded to the C−H stretching vibration of C = C − H on the fat chain [33]. The peak at 1747 cm^−1^ corresponded to the carbonyl groups on glyceride, and the peak at 1655 cm^−1^ indicated the presence of unsaturated carbon–carbon double bonds in corn oil. From the spectrum of ACoO, it was revealed that the peak at 3010 cm^−1^ disappeared after acrylation, indicating that unsaturated double bonds participated in the reaction. At the same time, a new peak appeared at 1724 cm^−1^, corresponding to the C = O stretching vibration in acrylate groups after grafting. The peaks at 1637 and 1619 cm^−1^ corresponded to the C = C stretching vibration in acrylate groups, which were much stronger than the peak at 1655 cm^−1^, corresponding to the unsaturated double bonds in CoO [34]. The peak at 1406 cm^−1^ corresponded to the CH_2_ vibration of CH_2_ = C in the acrylate groups [24,34]. The peaks at 1296 and 1272 cm^−1^ corresponded to the CH bending vibration of CH= in the acrylate groups. The peaks at 984 and 966 cm^−1^ corresponded to the in-plane rocking vibration of CH_2_= in the acrylate groups. The above analysis showed that the corn oil-based acrylate prepolymer (ACoO) was successfully synthesized by the grafting of acrylate to the unsaturated double bonds of corn oil.

**Figure 2:**
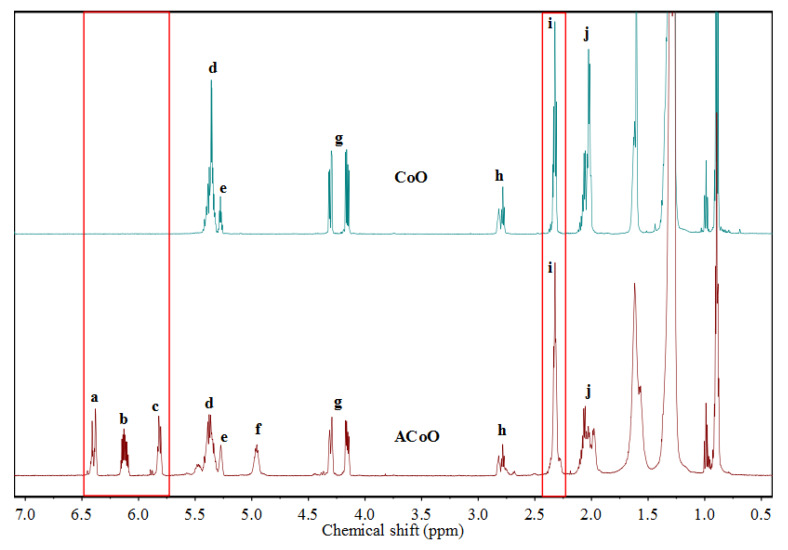
Proton nuclear magnetic resonance (^1^H NMR) spectra of CoO and ACoO. Note: The lowercase letters corresponded to the protons in Scheme 2.

The proton nuclear magnetic resonance (^1^H NMR) spectra of CoO and ACoO are shown in Figure 2. In the spectrum of CoO, the peaks at *H*_d_ (5.30–5.50 ppm) and *H*_e_ (5.25–5.30 ppm) corresponded to the protons bound to the unsaturated double bonds and the methine proton of the triglyceride, respectively [22], although these two peaks overlapped slightly. The peak at *H*_g_ (4.05–4.35 ppm) corresponded to the methylene protons of triglyceride [22]. The peak at *H*_h_ (2.70–2.75 ppm) corresponded to the methylene proton which sandwiched between two double bonds [35]. The peak at *H*_i_ (2.20–2.40 ppm) corresponded to the methylene protons adjacent to carbonyl groups, and it was selected as internal standard due to its good stability in the reaction and its obvious appearance in ^1^H NMR spectrum [22]. The peak at *H*_j_ (1.90–2.10 ppm) corresponded to the methylene protons adjacent to the double bonds [35]. Through normalization, it was found that the peak area ratio of *H*_i_ to *H*_g_ was 6:4, indicating that CoO contained a triglyceride structure. The double bond content in CoO could be determined by comparing the peak areas of *H*_d_ and *H*_i_. However, since there was a certain overlap between the peaks of *H*_d_ and *H*_e_, the peak area of *H*_d_ could be determined by subtracting the peak area of *H*_e_ from the total peak area. Due to its triglyceride structure, the peak area ratio of *H*_e_ to *H*_g_ was 1:4. The following formula can be used to calculate the number of double bonds contained in each CoO molecule [30]:

(1) $$DoublebondsperCoOmolecule=\frac{\left(A_{d,e}-A_{g}/4\right)/2}{A_{i}/6}$$

where *A_d,e_*, *A_g_*, and *A_i_* represent the peak area of *H*_d,e_, *H*_g_, and *H*_i_, respectively.

Each CoO molecule contained 4.36 double bonds.

In the ^1^H NMR spectrum of ACoO, the peaks at *H*_a_ (6.30–6.50 ppm), *H*_b_ (6.10–6.20 ppm), and *H*_c_ (5.75–5.90 ppm) corresponded to the three double bond protons on the acrylate groups, respectively (Scheme 2) [36]. The ratio of peak areas was about 1:1:1. The peaks at *H*_d_, *H*_h_, and *H*_j_ weakened significantly after the reaction. The newly emerging peak at *H*_f_ (4.90–5.00 ppm) corresponded to the methine proton attached to the acrylate groups [37]. These changes indicated the successful grafting of the acrylate to the unsaturated double bonds of CoO through acrylation to obtain the acrylate prepolymer ACoO. The grafting number can be calculated from the following formula [30]:

(2) $$Grafting\;number=\frac{\left(A_{a}+A_{b}+A_{c}\right)/3}{A_{i}/6}$$

where *A_a_*, *A_b_*, *A_c_*, and *A_i_* represent the peak area of *H*_a_, *H*_b_, *H*_c_, and *H*_i_, respectively.

The peaks at *H*_a_, *H*_b_, *H*_c_, and *H*_f_ became more obvious as the reaction proceeded, and the peaks at *H*_d_, *H*_h_, and *H*_j_ did not disappear completely, but became weaker, which revealed that the unsaturated double bonds of CoO did not react completely [38]. The acrylation grafting rate of ACoO can be calculated by the following formula:

(3) $$Grafting\;rate=\frac{Grafting\;number}{Double\;bonds\;per\;CoO\;molecule}\times 100\%$$

Table 1 shows the acrylate grafting number and acrylation grafting rate of different vegetable oil-based acrylate prepolymers. As the number of double bonds in the vegetable oils increased, the grafting number increased, but the grafting rate showed a downward trend. Even though the acrylate grafting number increased with the increase in double bonds due to the steric hindrance of the already grafted acrylate species, further acylation of the adjacent unsaturated double bonds would be restricted, leading to the lowering of the acylation rate [30].

### 3.2. Dynamic Mechanical Analysis (DMA)

**Figure 3:**
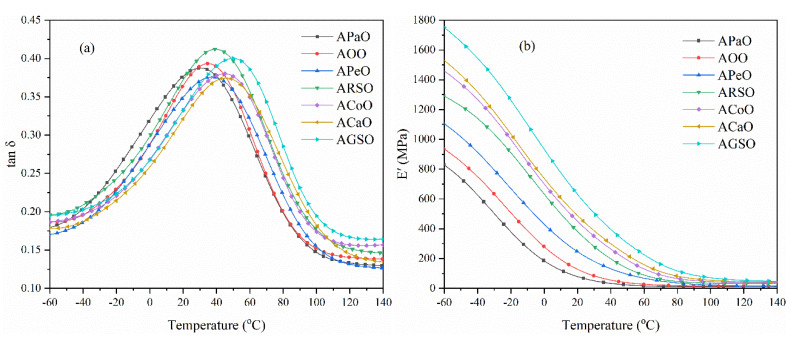
(**a**) Loss factor and (**b**) storage modulus of the UV-cured films.

The glass transition temperature (*T*_g_), storage modulus (*E′*), and cross-linking density (*v*_e_) of a series of UV-cured films were studied by dynamic mechanical analysis (DMA). Figure 3 shows the trend of the loss factor (tanδ) and storage modulus (*E′*) of the films with temperature. Table 2 lists the relevant *T*_g_, *E′,* and *v*_e_ data. Generally, the temperature at the peak of the tanδ curve is the *T*_g_ of the cured film. As shown in Figure 3b, in the low temperature region, all films showed a high *E′* value, and *E′* dropped rapidly with increasing temperature due to the glass transition. In this study, the vegetable oil-based prepolymers with a higher number of acrylate grafting had a higher double bond density, so it was easier to obtain a higher cross-linking density, so their storage modulus was increased. In addition, it can be seen from Figure 3a that all films only had one tanδ peak, indicating that the formulated systems had good compatibility between the different components. In order to further study the thermo-mechanical properties of the films, the cross-linking density (*v*_e_) of the films can be calculated by the following formula [18,36]:

(4) $$E^{\prime }=3RT{}^{\prime }\times v_{e}$$

where *T′* represents the absolute temperature of the films in the rubber state (*T*_g_ + 30 °C), *E′* represents the storage modulus of the films at *T′*, and *R* is the gas constant.

The cross-linking density (*v*_e_) values of different vegetable oil-based cured films are shown in Table 2. It was found that as the grafting number increased, the *v*_e_ value of these films increased from 2.0 × 10^3^ to 13.3 × 10^3^ mol·m^−3^, and the glass transition temperature *T*_g_ increased from 30.3 to 50.0 °C, which indicated that the films became harder. This is because the vegetable oil-based prepolymers with a high grafting number had a higher double bond density, and it was easier to obtain a higher cross-linking density.

### 3.3. Thermal Stability

**Figure 4:**
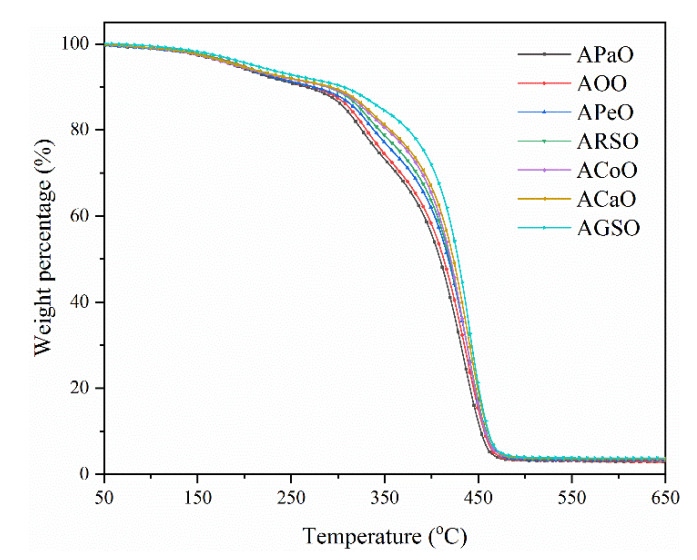
Thermo-gravimetric analysis (TGA) results of the UV-cured films.

The thermal decomposition curves of different vegetable oil-based cured films are shown in Figure 4. Char yield was measured at 650 °C. Table 3 lists the thermal decomposition temperature of all films at the weight loss of 10% (*T*_10%_) and 50% (*T*_50%_). Two degradation stages were demonstrated in the thermal decomposition curves. Under a nitrogen atmosphere, the films were relatively stable between 50 and 150 °C. The first degradation stage occurred between 150 and 350 °C, which was mainly due to the decomposition of the PI-1173 and residual raw materials. The second degradation stage occurred between 340 and 460 °C mainly due to the molecular chain decomposition and carbonization of the cross-linked polymers. In addition, as shown in Table 3, when the grafting number increased, the values of *T*_10%_, *T*_50%_, and char yield also increased, indicating that the vegetable oil-based prepolymers with a higher grafting number had a higher double bond density, and it was easier to obtain a higher cross-linking density, thereby improving the thermal stability of the films [36]. Overall, all cured films had excellent thermal stability, indicating that they could be used at high temperatures.

### 3.4. Mechanical Properties

**Figure 5:**
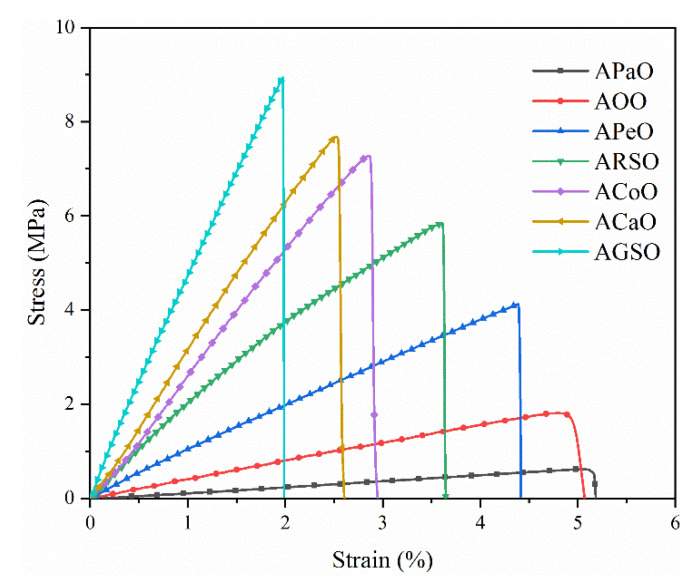
Stress–strain curves of the UV-cured films.

The stress–strain curves of different vegetable oil-based cured films are shown in Figure 5. Accordingly, Table 4 presents the data about their tensile strength, elongation at break, and Young’s modulus. The stress–strain curves of all cured films showed that the stress continued to increase with the increase of strain, and they broke before reaching the yield point, indicating their rigid characteristics. In addition, as the grafting number increased, the corresponding tensile strength increased from 0.62 to 8.94 MPa, and the Young’s modulus increased from 13.93 to 468.07 MPa, but the elongation at break decreased from 5.12 to 1.97%. The changing trend of mechanical properties can be explained by the inherent chemical composition and cross-linking density. In the cured systems, the increase in the grafting number resulted in an increase in the functionality of their structure, which was conducive to the formation of high cross-linking density and helped to increase the rigidity of the cured films, thereby enhancing the mechanical properties of the cured films [39,40].

In addition, the characteristics of different vegetable oil-based UV-cured films were compared. As shown in Table 5, at least two steps were needed to prepare the vegetable oil-based prepolymer in other work, while our work used a one-step method to synthesize the prepolymer, which greatly simplified the synthetic route. Furthermore, these UV-cured films exhibited properties comparable to other films including high *T*_g_, 50% thermal decomposition temperature, and tensile strength.

## 4. Conclusions

In this study, palm oil, olive oil, peanut oil, rapeseed oil, corn oil, canola oil, and grapeseed oil were used as raw materials to synthesize different vegetable oil-based acrylate prepolymers by one-step acrylation. The as-prepared vegetable oil-based acrylate prepolymers, PUA-2665, TMPTA, and PI-1173 were mixed thoroughly to prepare the UV-curable films. The results revealed that as the number of double bonds in vegetable oil increased, the grafting number corresponding to the products increased from 1.13 to 2.17, and the grafting rate decreased from 66.47 to 47.90%. This was because the increased double bonds could speed up the reaction rate, whereas the branched acrylic groups had steric hindrance effect, preventing adjacent double bonds from participating in the reaction. Furthermore, the increased grafting number led to an increase in their functionality, which increased the cross-linking density from 2.0 × 10^3^ to 13.3 × 10^3^ mol·m^−3^, thereby enhancing the thermal stability and mechanical properties of the cured films. Glass transition temperature increased from 30.3 to 50.0 °C, 50% thermal decomposition temperature increased from 408.9 to 428.3 °C, and tensile strength increased from 0.62 to 8.94 MPa. A series of cured films from soft to tough were prepared by using different vegetable oils, among which grapeseed oil-based UV-cured film had the best thermal (glass transition temperature) and mechanical (tensile strength, Young’s modulus) properties. The adjustable properties of vegetable oil-based prepolymers make them a potential candidate to be used in UV-curable coatings. This work summarizes the inherent relationship between the structure of vegetable oils and the properties of UV-cured films, and it provides guidance for the synthesis of vegetable oil-based acrylate prepolymers and their applications in UV-curable coatings.

## Figures and Tables

**Table 1:** Grafting number and grafting rate of different vegetable oil-based acrylate prepolymers.

Samples	Double Bonds	Grafting Number	Grafting Rate (%)
APaO	1.70	1.13	66.47
AOO	2.82	1.59	56.38
APeO	3.48	1.72	49.43
ARSO	3.81	1.85	48.56
ACoO	4.36	2.04	46.79
ACaO	4.47	2.06	46.09
AGSO	4.53	2.17	47.90

**Table 2:** Dynamic mechanical properties and cross-linking density of the UV-cured films.

Samples	*T*_g_ (°C)	*E′_25_* (MPa)	*E′* at *T*_g_ + 30 °C (MPa)	*ν*_e_ (×10^3^ mol·m^−3^)
APaO	30.3	63.6	16.8	2.0
AOO	35.5	103.2	24.4	2.9
APeO	38.3	209.2	53.9	6.3
ARSO	40.1	326.9	68.4	8.0
ACoO	45.0	397.5	74.7	8.6
ACaO	46.4	435.4	92.0	10.6
AGSO	50.0	565.8	117.3	13.3

**Table 3:** Thermal stability of the UV-cured films.

Samples	*T*_10%_ (°C)	*T*_50%_ (°C)	Char Yield (%)
APaO	265.9	408.9	2.47
AOO	270.6	412.0	2.61
APeO	279.0	417.3	2.73
ARSO	287.5	418.2	3.15
ACoO	290.7	420.5	3.39
ACaO	291.7	423.4	3.54
AGSO	304.4	428.3	3.74

**Table 4:** Mechanical properties of the UV-cured films.

Samples	Tensile Strength (MPa)	Elongation at Break (%)	Young’s Modulus (MPa)
APaO	0.62 ± 0.08	5.12 ± 0.61	13.93 ± 3.37
AOO	1.75 ± 0.16	4.91 ± 0.74	37.15 ± 8.61
APeO	4.10 ± 0.21	4.39 ± 0.13	94.07 ± 13.87
ARSO	5.81 ± 0.52	3.61 ± 0.67	169.82 ± 28.25
ACoO	7.26 ± 1.07	2.87 ± 0.54	259.72 ± 24.61
ACaO	7.67 ± 0.93	2.54 ± 0.29	301.85 ± 23.93
AGSO	8.94 ± 1.10	1.97 ± 0.09	468.07 ± 31.06

**Table 5:** Comparison of the vegetable oil-based UV-curable prepolymers and the properties of the resultant cured films.

Vegetable Oils	Number of Steps	Functionality	*T*_g_ (°C)	*T*_50%_ (°C)	Tensile Strength (MPa)	References
Castor oil	2	3	32.0–72.1	416.0–428.7	8.15–12.32	[22]
Tung oil	2	6	85.7–123.7	440.3–465.9	10.72–18.07	[18]
Palm oil	2	3	115.5–119.6	440.3–444.0	5.2–6.2	[41]
Soybean and camelina oil	2	2.5–3.3	43.8–67.7	415	8.9–17.0	[42]
A range of vegetable oils	1	1.13–2.17	30.3–50.0	408.9–428.3	0.62–8.94	Present
